# COVID Sniffer Dogs: Technical and Ethical Concerns

**DOI:** 10.3389/fvets.2021.669712

**Published:** 2021-06-21

**Authors:** Biagio D'Aniello, Claudia Pinelli, Mario Varcamonti, Marcello Rendine, Pietro Lombardi, Anna Scandurra

**Affiliations:** ^1^Department of Biology, University of Naples Federico II, Naples, Italy; ^2^Department of Environmental, Biological and Pharmaceutical Sciences and Technologies, University of Campania “L. Vanvitelli”, Caserta, Italy; ^3^Department of Clinical and Experimental Medicine, University of Foggia, Foggia, Italy; ^4^Department of Veterinary Medicine and Animal Production, University of Naples Federico II, Naples, Italy

**Keywords:** SARS-CoV-2, COVID-19, dogs, volatile organic compounds, detection dogs

Hindu medicine showed us that some diseases can alter a humans' scent. Some diseases emit specific volatile organic compounds (VOCs) from exudates, which can be used as a diagnostic tool (1). Recently, occidental culture has started to identify diseases through olfaction, including smallpox (2). Some studies have underlined that humans can identify individuals with bacteria-derived endotoxins through sweat, considering the smell of the sweat unpleasant (3). This olfactory cue could activate a social avoidance, helping to stay far from infected people, thus limiting the contagion in humans, as well as in other animals (4). A human's sense of smell is probably underestimated (5), but it is undoubtedly not capable enough to identify pathogens in people with subliminal changes in odor.

While humans may not be able to detect these subtle odor changes, dogs are capable. They can smell different molecules from the human body released during some emotional states (6–9), and they can be trained to give specific signals when identifying an olfactory signature (i.e., alert signal). Dogs are currently used as special sensors to detect VOCs (10). They have been successfully trained to detect several metabolic conditions and diseases in humans, including hypoglycemia and hyperglycemia (11, 12), epileptic seizures (13), cancers (14), and bacterial and viral infections (15). However, despite the undoubted individual abilities of trained animals, we are still far from a detailed understanding of what exactly the dog responds to and the possibility of generalizing certain abilities to all dogs.

It has been shown in recent studies that dogs can detect people infected with SARS-CoV-2 (16). The use of dogs for this purpose could be critical during emergencies as well as when diagnostic technologies require a long time to be applied. Thus, the dog could be the best detection device in these cases (17) as it represents a faster method of identifying infected people by a non-invasive procedure. Moreover, the use of dogs would allow operators to avoid contact with infected individuals. Though medical detection dogs could be very expensive to train (18), they could test hundreds of people per day, reducing reagent costs.

In this opinion article, we questioned whether there is sufficient scientific support to justify the training and use of dogs as biological detector systems for SARS-CoV-2 in reasonable time frames and safety. To warrant human and dog health, we analyzed the recent scientific literature and discussed different technical and ethical problems with the involvement of dogs for detecting SARS-CoV-2 in people: the “context-shift effect,” the overlap of VOC profiles in different diseases and odors that may occasionally co-occur, the procedure to collect samples, and the possible role of the animals as vectors in a zoonotic scenario.

The SARS-CoV-2 pandemic stimulated scientific inquiries on the ability of dogs to recognize the smell of infected human samples. Grandjean et al. (19) trained six detection dogs in recognizing the smell of SARS-CoV-2-infected people by using armpit sweat samples. Their proof-of-concept study concluded that dogs can detect subjects with SARS-CoV-2 with a very high success rate (ranging between 76 and 100%). Another scientific study tested the effectiveness of dogs in distinguishing saliva samples and tracheobronchial secretions from SARS-CoV-2 patients with clinical symptoms (20). The authors demonstrated that dogs were able to master the task with high rates of sensitivity (average 82.63%) and specificity (average 96.35%) after a week of training. A third study trained dogs to recognize SARS-CoV-2-infected people from respiratory samples (i.e., saliva, nasopharyngeal swabs or aspirates, and tracheal aspirates) obtained from subjects with mild, moderate, or severe symptomatology (21). Also, in this case, the results were very promising, with the testing procedure reaching 95.5% sensitivity and 99.6% specificity. These studies showed the high efficacy of dogs in recognizing infected people, thus making them useful tools for a very quick screening in crowded places, such as airports and schools. The scientific results have aroused considerable popular enthusiasm, as pointed out by the media, which consider the dogs easy to train and to operate in work contexts.

Besides these advantages, several technical and ethical concerns may occur by using the dogs as sensors of subjects infected by SARS-CoV-2, which seem poorly considered by media and sometimes underestimated in scientific papers. The scientific studies, while demonstrating the effectiveness of the detection dogs for the SARS-CoV-2, have been very cautious in suggesting the use of their results to train dogs for operative purposes, pointing out several pitfalls. Despite that, it seems that the unsolved issues have not reached the practitioners, and therefore, several canine centers are training and using dogs to detect SARS-CoV-2 infected subjects. According to the present scientific evidence, we believe that this approach is not justified at the moment, for several technical and ethical concerns.

The major problem is that no data are available on the performance of dogs in the field as the application of the models in the laboratory studies has not been scientifically tested in work contexts, thus making the effectiveness of dogs questionable. When animals that learned to perform a behavior under a stimulus in a context are moved to a new context, the performance generally drops, which is known as the “context-shift effect” (22) and maybe reflects the loss of information acquired to achieve the goal. This effect has been observed in dogs highly trained for detecting explosives (23). Additionally, a study on detection dogs for lung cancer patients found that by shifting from a hospital to another location, the dogs' performance was significantly reduced, decreasing sensitivity and increasing the occurrence of false positives (24).

The recognition of the VOCs produced by the viral infection presents some difficulties due to the biology of the infection processes, which induce in the host the production of additional VOCs. Generally, viruses do not have their own metabolism; thus, the elicited VOCs could only arise from the inflammatory responses of the infected host (25). It is unknown if SARS-CoV-2 induces changes in VOCs sharing no commonalities with other inflammatory diseases and whether new variants have the same effect in terms of odor changes. Some of the VOCs produced in a single cell line of the infective viruses H9N2, H6N2, and H1N1 appeared selective for each virus, but a plethora of several other non-specific VOCs were present (26). A study on breath analysis using multi-capillary column-ion mobility spectrometry showed that it is possible to discriminate between influenza A and SARS-CoV-2 infections based on the different VOC profiles, although specific VOCs were not identified (27). The authors suggested that dogs could be used to successfully discriminate SARS-CoV-2 infection from other infective diseases. It has been demonstrated that dogs can discriminate VOCs caused by similar virus infections, such as bovine viral diarrhea virus, bovine herpesvirus, and bovine parainfluenza virus (28). Nevertheless, based on previous studies, it is not possible to know for sure if dogs could be confused when detecting between SARS-CoV-2 variants and between variants and other viruses.

In addition to the VOC discrimination problems in infected individuals, another confounding factor could be represented by the overlap of biochemical signals. This phenomenon could confuse dogs, decreasing their detection performance, although the specific combination and concentration of the relevant VOCs may be sufficient for a dog to identify a positive sample. The problem becomes more complex when examining VOCs from the human body while keeping control of the dog's conditioning, which is very important when trying to reduce false positives. For example, two dogs in the bioRxiv version of Grandjean et al. (29) study marked positive a sample from a negative woman that was around the ovulation period, when the luteinizing hormone (LH) peaks. Another study reported that SARS-CoV-2-infected men may show increased levels of LH (30), which makes it plausible to assume that dogs could be conditioned on the metabolic change triggered from the LH instead of that elicited from the virus.

An important factor to be carefully analyzed is the collection and the preparation of the experimental samples for the dog's training. Studies testing the skill of dogs to recognize SARS-CoV-2-infected biological samples worked with a relatively small number of independent and single samples (19–21). This procedure cannot exclude that dogs could memorize the odor of the person, rather than that elicited by the SARS-CoV-2 infection. Indeed, the scientific literature recommends avoiding repeated presentation of samples from the same donors to detection dogs (31).

In the available literature, the samples were collected from symptomatic people; thus, it is unclear whether dogs would alert on samples from asymptomatic individuals. Of course, this is the most important aspect when aiming to identify possible virus spreaders. More research is therefore needed to verify whether dogs could identify asymptomatic and pre-symptomatic individuals. A paper published in August 2020 (32) stated that they were testing dogs to identify asymptomatic people, but the results of this project are not yet available, as well as in the case of Vesga et al. (21).

Beyond the technical aspects of using dogs as sensors, there are also ethical concerns related to the zoonotic transmission of SARS-CoV-2. To date, the bat origin of SARS-CoV-2 remains the most probable cause of the pandemic in humans (33), and several natural, farmed, pet, and wild animal species have been found infected (34). Minks can have severe symptoms from the infection, and they can die of pneumonia (35). SARS-CoV-2-specific antibodies were not found in 35 animal species tested using double-antigen sandwich ELISA, including dogs and cats (36), but they were detected in dogs and cats by using plaque reduction neutralization tests (37, 38). Dogs were significantly more likely to test positive for SARS-CoV-2-neutralizing antibodies if living in households with infected humans (38, 39), and apart from some negative reports (40), many studies agree that dogs could become infected by humans, although they do not report symptoms from the SARS-CoV-2 infection (41–46). On the other hand, even in healthy humans, most cases were relatively mild or asymptomatic, but older patients and comorbidities could result in severe cases (47). Currently, only a handful of healthy dogs have been studied, and no studies verified the effect of SARS-CoV-2 in old dogs or dogs with other diseases. A study with an artificial infection on five 3-month-old beagles found low susceptibility to SARS-CoV-2 (48), but once again, the samples tested were limited. Should SARS-CoV-2 evolve to be a significant clinical infection in dogs is at the moment unknown. The angiotensin-converting enzyme type 2 receptors (the entry point into cells for some coronaviruses, including SARS-CoV-2) of dogs are very similar to those of humans, with an identity of 83% (49), which does not discharge the risk that dogs could serve as an intermediate host (44, 50). Viruses are well-known to evolve in real time, especially when under immunological pressure, to ease their transmission between humans (51) and from animals to humans (52). A new variant found in humans arose in minks (53). We cannot exclude that new variants in humans may become more infectious for dogs and vice versa, nor can we exclude that new variants in dogs could become more efficient by increasing intraspecies and interspecies transmission. In our opinion, there are currently insufficient results to make sure that dogs could not be or become a reservoir species, whereby we should be more cautious before deliberately exposing dogs to SARS-CoV-2. One of the most important strategies for limiting the pandemic is to identify the potential virus reservoir to prevent any spillover effects, certainly not to facilitate a potential new reservoir species. There is evidence that experimentally infected cats (37, 48, 54), hamsters (55, 56), ferrets (48, 57), and minks (35) may spread SARS-CoV-2, while pigs and some poultry species do not (48, 58, 59). In some cases, the situation is worrying as bilateral transmission between humans and animals has been proved [i.e., minks (60, 61)]. Some studies underlined that there is currently no evidence that infected dogs could be a source of infection for humans (37, 46, 62, 63), although further epidemiological investigations are requested before reaching a definitive conclusion (63). Actually, as a precautionary principle, the fact that there is no scientific evidence does not mean that it could not happen. Some studies have not excluded that dogs could play a role in spreading the virus to other dogs and other animals, including humans (41, 43). The uncertainty of classifying dogs as non-spreaders violates the rules of infection prevention and control.

The authors of the studies that tested dogs to detect people infected with SARS-CoV-2 have been very careful to avoid the exposure of dogs to infections (19, 21, 43), and indeed, in their experimental setting, there was no risk to dogs. However, the laboratory conditions are different from those of the operational work. The fact that SARS-CoV-2 is absent from human sweat (64, 65) may make dogs safe in laboratory tests, but not in a naturalistic scenario where control is more difficult. Although anatomical sites such as armpits are protected by contamination, the part should be uncovered by the hands of the potentially infected subject, which does not warrant sterility, especially when the person is requested to pick up the sample on their own. Fathizadeh et al. (66) collected forehead sweat samples from positive people, and even after disinfecting the skin with 70% ethanol, two positive cases were found in up to 25 infected patients. The authors concluded that although patients' sweat does not contain SARS-CoV-2, it can be easily contaminated. In the study by Jendrny et al. (20), the patient samples were inactivated after incubation for 70–72 h with a chemical compound (i.e., propiolactone) to inactivate the virus. This procedure, while eliminating the risk of contagion, makes faster use of dogs impractical.

To summarize, we reported some suggestions to the problems pointed out in this opinion. Dogs' effectiveness should be tested in different testing environments and naturalistic scenarios to avoid the context-shift effect. It should be a priority to delineate the VOC profiles of the samples of infected people, as collected, using headspace solid-phase microextraction combined with gas chromatography–mass spectrometry, before utilizing them for training the dogs. In the same way, the VOC profiles of the samples should be delineated from non-diseased subjects (67). This procedure would allow comparison of symptomatic and asymptomatic subjects, age classes, sexes, and different parts of the body sample. Although dogs can be trained in the absence of such information, this technical approach is important to allow researchers and stakeholders to control the training at best, thus reaching more suitable performances. The use of VOC-free support materials is recommended to prevent contamination in the results. In the absence of VOC-free gauzes and tubes, these should be pretreated to remove VOC contaminants as described by Cardinali et al. (68). To rule out interindividual differences in body odor, exudates from a large number of different individuals should be collected and mixed (7), or at least different samples should be used for training and testing procedures. To further minimize the chance of dogs memorizing odors from individuals, they should also be trained with the exudates of the same subject collected during both the infective and healthy phases. In that case, it would be necessary to know how long individuals can maintain the odor, especially if matched samples are used. From the reviewed literature, we have a very low chance of SARS-CoV-2 contagion by interacting with our pet dogs. However, it is undoubtful that greater awareness is needed for understanding the possible involvement of dogs in virus hosting and spreading, using a broader vision in the One Health approach. We are not proposing to completely abandon the sniffing dog strategy. We advocate the precautionary principle and highlight the need for further scientific studies addressing the concerns outlined in this opinion paper before claiming that we can safely use and train dogs effectively to detect SARS-CoV-2-infected people. Particularly, developing a vaccine for dogs could help mitigate the underlined ethical concerns. However, this procedure does not warrant that dogs could serve as a reservoir for the SARS-CoV-2 and develop new variants.

Only after having passed all these scientific steps can we start using dogs in work contexts with more reasonable effectiveness.

## Author Contributions

BD'A, CP, MV, MR, PL, and AS have made a substantial, direct and intellectual contribution to the work, and approved it for publication.

## Conflict of Interest

The authors declare that the research was conducted in the absence of any commercial or financial relationships that could be construed as a potential conflict of interest.
